# Draft Genome Sequence of the Marine Bacterium *Alteromonas* sp. Strain KS69

**DOI:** 10.1128/MRA.00022-19

**Published:** 2019-01-31

**Authors:** Kaitlin J. Schulz, Brianna E. Arruda, Adam S. Bitzer, Mark W. Silby

**Affiliations:** a Department of Biology, University of Massachusetts Dartmouth, Dartmouth, Massachusetts, USA; Georgia Institute of Technology

## Abstract

Alteromonas spp. are Gram-negative, aerobic, marine bacteria. Here, we report the draft genome sequence of Alteromonas sp. strain KS69, isolated from Narragansett Bay deep water samples. Unpublished preliminary data suggest that KS69 reduces expression of the 3-oxo-C12-HSL-dependent, virulence-associated gene *lasB* of Pseudomonas aeruginosa PAO1, suggesting that it produces a quorum sensing inhibitor.

## ANNOUNCEMENT

The clinically relevant pathogen Pseudomonas aeruginosa uses quorum sensing (QS) initiated by *N*-acyl homoserine lactone (AHL) autoinducers (1) to regulate expression of virulence factors. QS inhibition is of interest as an antivirulence strategy that may be useful alongside antibiotics for treating infections. Toward this goal, we have been bioprospecting for marine bacteria positive for QS inhibition in Narragansett Bay, Rhode Island. Seawater samples were plated on Difco marine agar 2216 and incubated at 20°C. Several isolates tested for QS disruption using the QS reporter plasmid pSB1142, which responds to the P. aeruginosa AHL 3-oxo-C_12_-HSL (2), were positive for QS inhibition (unpublished data). Among these, an isolate (KS69) was identified as an Alteromonas sp. by analysis of the 16S rRNA gene using the Ribosomal Database Project (RDP) Classifier (3). Alteromonas spp. are Gram-negative, aerobic, motile marine bacteria known for secondary metabolite production and hydrocarbon degradation (4). Some Alteromonas species are capable of quorum quenching (QQ), the inhibition of QS by signal molecule destruction, using acylases which separate the acyl side chains from the lactone ring of AHL (5, 6).

The Wizard genomic DNA purification kit (Promega) was used to extract total DNA from Alteromonas sp. strain KS69, after which the library for sequencing was prepared using the Nextera XT DNA library preparation kit (Illumina). DNA sequencing was carried out by the Tufts University Genomics Core on an Illumina MiSeq sequencer. Reads that passed Illumina quality control (pipeline 1.9) were imported into CLC Genomics Workbench 10.1.1 (Qiagen). These had a mean quality score of 34.18 with 84.85% greater than Q30. The 2,495,192 2 × 250-bp paired-end reads were assembled into 84 contigs with an estimated 171-fold coverage, using CLC Genomics Workbench with the minimum contig size set to 1,000 bp and word size set to 22. Contig size ranges from 1,020 to 538,706 bp with an *N*_50_ scaffold size of 184,109 bp. Total size for combined contigs was 4,882,533 bp with a G+C content of 43.6%. Annotation was added by the NCBI Prokaryotic Genome Annotation Pipeline (PGAP) (6). The genome is predicted to contain 4,177 protein-coding genes and 54 tRNA genes. The annotation identified 6 rRNA genes.

In addition to QQ via acylase production by Alteromonas spp., QQ could occur via lactonase production (5, 7). We searched the KS69 genome using TBLASTN for putative AHL acylase or lactonase genes using query sequences collated by Muras et al. (8). Two lactonase query sequences returned weak matches with predicted hydrolase enzymes (identities less than 35%, query coverage less than 60%). Most acylase query sequences returned weak matches with coding sequences annotated as acylase family proteins, while one returned matches with hydrolases. Matches with the putative acylase C7Y69_07810 ranged from 19.1% to 48.9% identity (one hit). Matches with the putative acylase C7Y69_18180 were limited to less than 41% coverage, with no matches having greater than 32.5% identity. All matches with putative hydrolases had less than 28% identity. These results do not exclude the possibility of KS69 encoding an AHL-degrading enzyme, but do not provide strong support for enzymatic degradation of AHLs. Experimental approaches supported by the genome sequence will be required to determine the basis of quorum sensing inhibition by KS69.

### Data availability.

This whole-genome shotgun sequencing project has been deposited in DDBJ/ENA/GenBank under accession number PYHU00000000. The version described in this paper is version PYHU10000000. Raw sequence reads have been deposited in the NCBI Sequence Read Archive under accession number PRJNA439351.
